# Supplementary material for: In Silico Analyses of the Role of Codon Usage at the Hemagglutinin Cleavage Site in Highly Pathogenic Avian Influenza Genesis
Source: Viruses. 2022 Jun 21;14(7):1352. doi: 10.3390/v14071352 (PMC9316147; doi:10.3390/v14071352)
Supplement: Supplementary file 1 [file viruses-14-01352-s001.zip › Supplementary File 1.pdf]

viruses

MDPI

Supplementary Figures

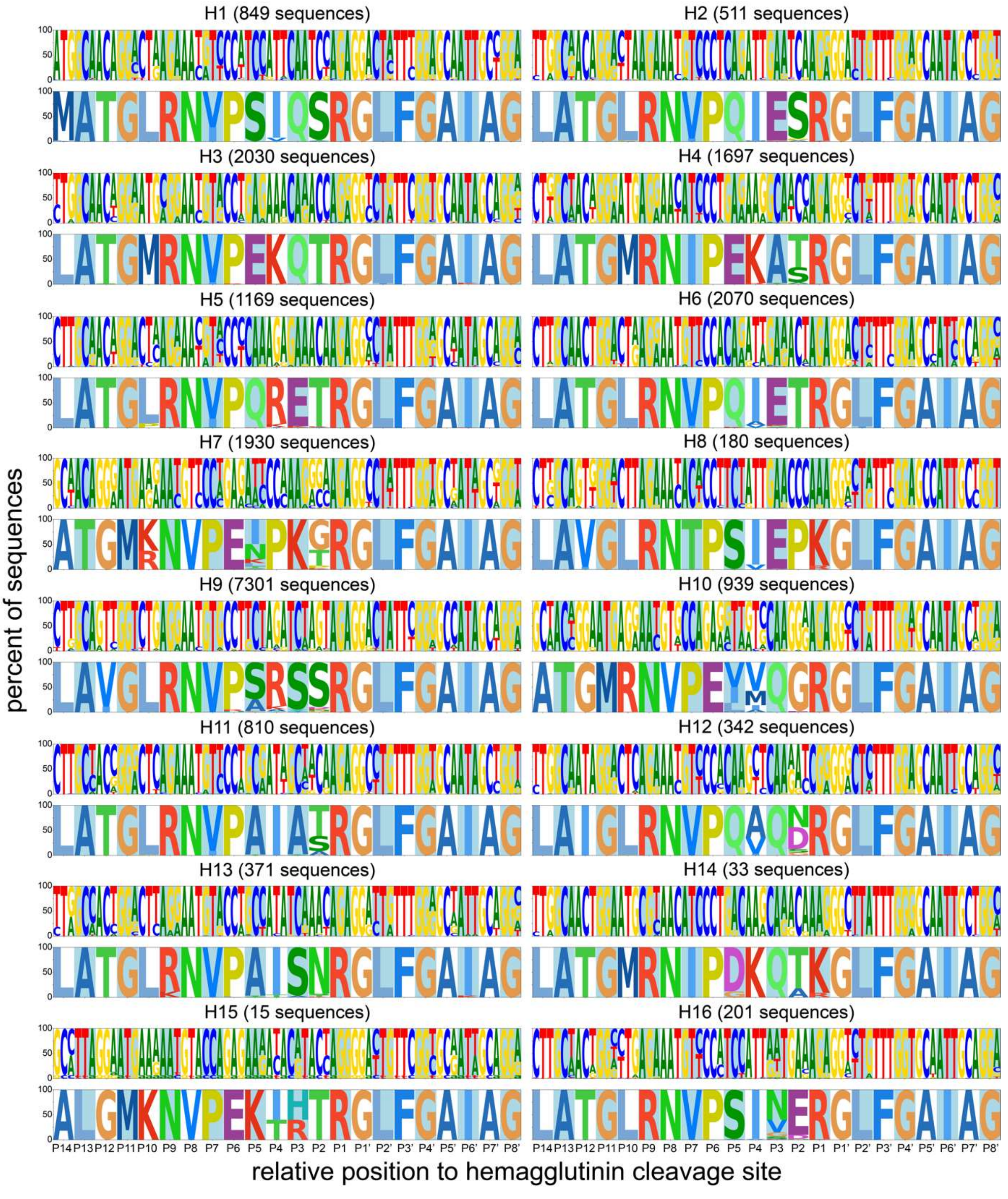

Figure S1. Sequence logos of nucleotide and amino acid composition of HA cleavage site regions (P14 to P8′, P1 position corresponding to the C-terminus of HA1) of different subtypes. For each HA subtype, the number of sequences used to generate the logo is indicated. Letter sizes are directly proportional to the percentage of sequences presenting a given character, as indicated by the axes on the left side of the logos.

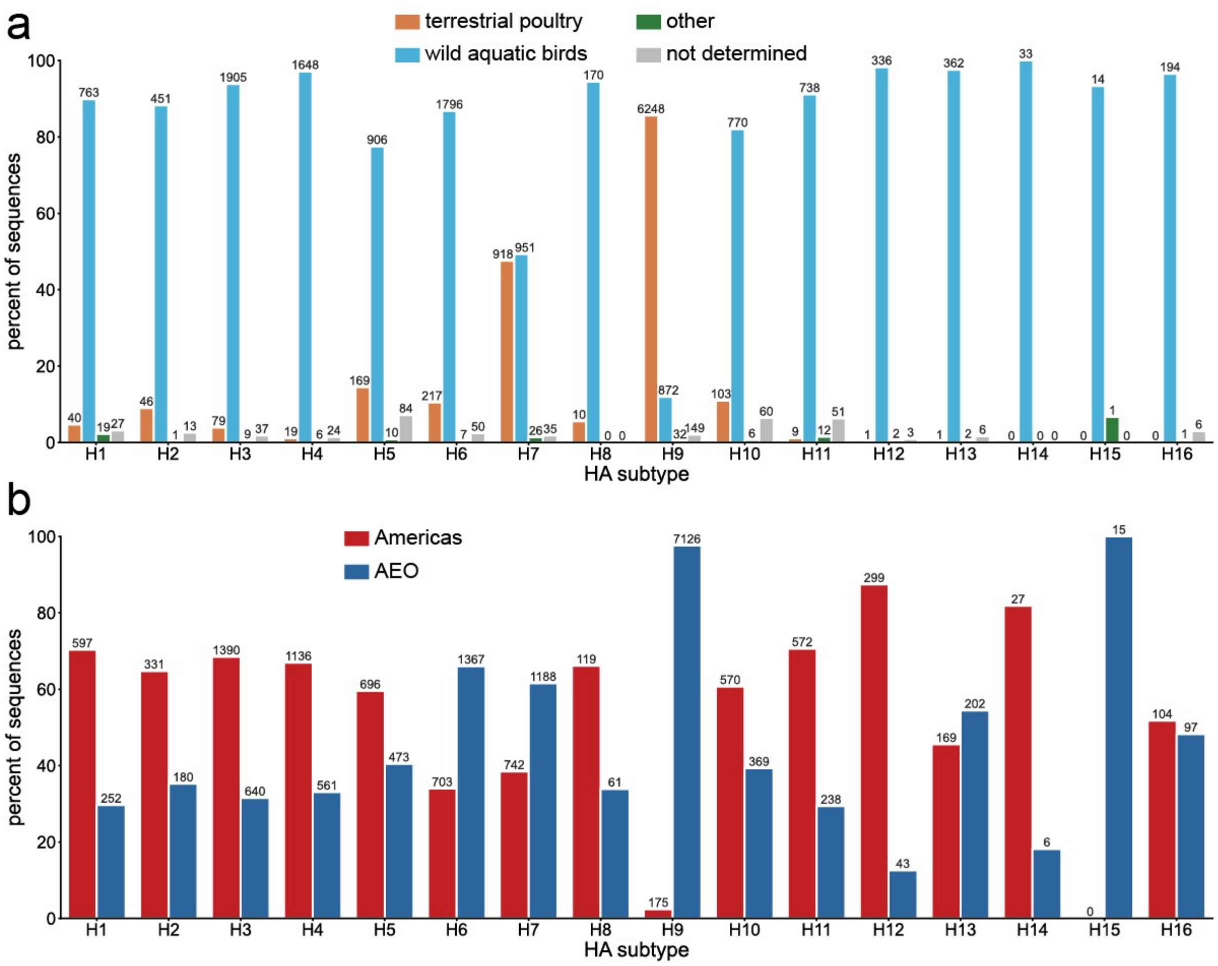

Figure S2. Virus species and region of isolation of HA sequences in the dataset. (**a**) Distribution per species category. Sequences were sorted into four species groups (terrestrial poultry, wild aquatic birds, others and not determined) and the percentage of sequences corresponding to each group was plotted for each HA subtype. The number of sequences in each group is indicated above each corresponding bar. Colors correspond to the legend above the graph. (**b**) Distribution per region of isolation. North and South America were grouped into the Americas; the rest of the world into Africa-Eurasia-Oceania (AEO), using the same representation as in (**a**).

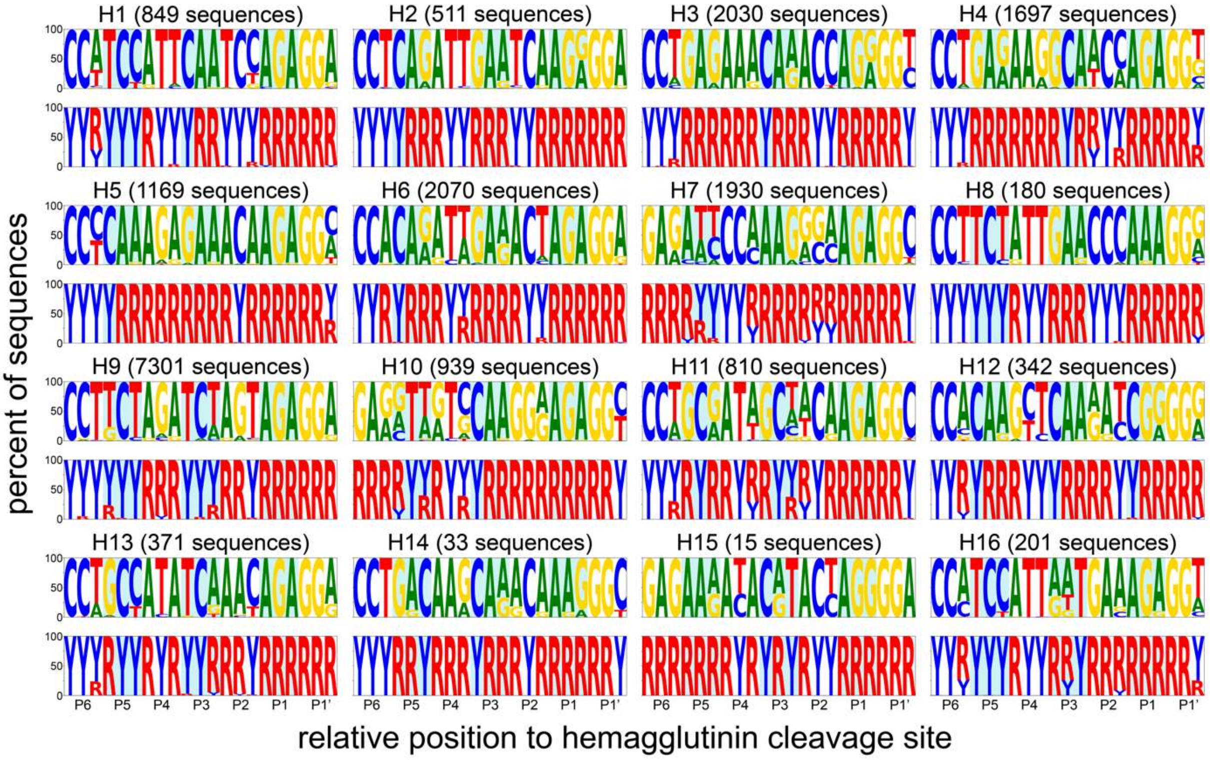

Figure S4. Sequence logos of nucleotide and purine (R)/pyrimidine (Y) composition of HA cleavage site regions (P6 to P1′, P1 position corresponding to the C-terminus of HA1) of different subtypes. For each HA subtype, the number of sequences used to generate the logo is indicated. Letter sizes are directly proportional to the percentage of sequences presenting a given character, as indicated by the axes on the left side of the logos.
