# Supplementary material for: In Silico Analyses of the Role of Codon Usage at the Hemagglutinin Cleavage Site in Highly Pathogenic Avian Influenza Genesis
Source: Viruses. 2022 Jun 21;14(7):1352. doi: 10.3390/v14071352 (PMC9316147; doi:10.3390/v14071352)
Supplement: Supplementary file 1 [file viruses-14-01352-s001.zip › Supplementary File 2.html]

Home
Figure S3a: number of adenines
Figure S3b: longest adenine stretch
Figure S3c: number of purines
Figure S3d: longest purine stretch
Figure S3e: substitutions to tribasic site
Figure S3f: substitutions to adenine stretch

all poultry/wild bird sequences
split by region of isolation
split by species of isolation
split by region and species of isolation

all poultry/wild bird sequences
split by region of isolation
split by species of isolation
split by region and species of isolation

all poultry/wild bird sequences
split by region of isolation
split by species of isolation
split by region and species of isolation

all poultry/wild bird sequences
split by region of isolation
split by species of isolation
split by region and species of isolation

all poultry/wild bird sequences
split by region of isolation
split by species of isolation
split by region and species of isolation

all poultry/wild bird sequences
split by region of isolation
split by species of isolation
split by region and species of isolation


## Supplementary Figure S3 for Funk et al. 2022

  
This figure is intended to highlight differences observed between sequences isolated from terrestrial poultry and aquatic wild birds. It therefore only represents the data obtained for sequences which could be conclusively sorted into these two species categories (see Table S2). Sequences sorted into the other or not determined categories are not represented in these graphs.  
  
Use the tabs above to navigate between the different analyses.  
  
Each analysis can be split by region, species or both by using the second row of tabs. For Figure S3f, the required A-threshold can be varied using the buttons in the top left corner. When split, the data is normalized by subtype as well as region, species or region and species, i.e., so that the sum of each stack of bars adds up to 100%  
  
Hover over a bar for more detailed information, such as the number of sequences it represents. Bar labels are displayed for bars representing fewer than 10 sequences, but over 50% of the data to highlight groups for which only a low number of sequences were available.  
  
All figures use the same color scheme: red for sequences isolated in the Americas, blue for sequences isolated in the Africa-Eurasia-Oceania (AEO) region using light colors for sequences isolated from terrestrial poultry and dark colors for sequences isolated from wild aquatic birds.  
  
If the graphs do not fill the window, try resizing it.  
  
**Figure S3a: Number of adenines in the P4 to P1 HA cleavage site region for each subtype.**  
The number of adenines in the 4 codons preceding the HA cleavage site was counted in each sequence.  
  
**Figure S3b: Length of the longest adenine stretch in the P4 to P1 HA cleavage site region for each subtype.**  
The longest stretch of adenines in the 4 codons preceding the HA cleavage site was identified in each sequence.  
  
**Figure S3c: Number of purines in the P4 to P1 HA cleavage site region for each subtype.**  
The number of purines (adenine or guanine) in the 4 codons preceding the HA cleavage site was counted in each sequence.  
  
**Figure S3d: Length of the longest purine stretch in the P4 to P1 HA cleavage site region for each subtype.**  
The longest stretch of purines (adenine or guanine) in the 4 codons preceding the HA cleavage site was identified in each sequence.  
  
**Figure S3d: Number of substitutions required in sequences from each subtype to obtain a tribasic cleavage site via substitutions.**  
For each sequence, the number of substitutions required to obtain an arginine in P1 and two other basic amino acids (lysine and arginine only) in P4 to P2 was determined. Only AGG and AGA codons were allowed for arginine, as only those are observed in HPAIVs in nature.  
  
**Figure S3f: Number of substitutions required to increase adenine stretch length in the P4 to P1 region using only codons found in the dataset at each given position in each subtype.**  
For each sequence, the number of substitutions required to obtain an adenine stretch of at least a given threshold is shown. An empty graph denotes the impossibility to obtain an adenine stretch of the indicated length using only codons observed in nature.


adenine threshold length:
3
4
5
6
7
8
9
10

adenine threshold length:
3
4
5
6
7
8
9
10

adenine threshold length:
3
4
5
6
7
8
9
10

adenine threshold length:
3
4
5
6
7
8
9
10
